# Enhancing the public health academic’s role in community engagement: building trusting relationships through support service delivery

**DOI:** 10.3389/fpubh.2025.1621156

**Published:** 2025-07-23

**Authors:** Angela Carman, Ashley Grospitch, Mary Elizabeth Pendergrass

**Affiliations:** University of Kentucky College of Public Health, Lexington, KY, United States

**Keywords:** community engagement, public health, academia, support service delivery, community health

## Abstract

Community engagement processes, specifically when initiated or led by public health researchers/academics often take the form of “outreach,” wherein the academic contacts a community experiencing a public health problem to perform a research study about the problem, or “consultation,” where the academic content expert is called on by a community to share what is known about a particular public health problem. Both of these forms of community engagement and those involving the public health academic/researcher have the potential to provide elements of community health improvement to the citizens in the community. Consider public health academic approaches to community engagement grounded in research models such as Community-Based Participatory Research, Implementation Research, and Team Science. Each of these models and strategies through which public health academics engage with communities have been widely used with many documented successful conclusions. However, based on work by the authors in communities across Kentucky over the past 10 + years, the possibility exists that specifically in rural communities with multiple public health issues and the frequent existence of a fractured public health infrastructure, another form of community engagement is needed for even better health improvement results. Although many Local Health Departments across the United States serve rural communities, only a small proportion of their directors have a formal public health education. With the increasing public health concerns of many areas, specifically those in rural areas like the majority of communities in Kentucky, the strain on the LHD staff and members of their community partner systems is great. From this perspective, the authors propose that the missing link in the previously discussed community engagement methods of outreach, consultation, and collaboration is support service delivery. Support service delivery, as we propose, would be delivered in a similar mechanism to preceptor guided training in certain health disciplines in which the expert demonstrates, teaches, and supports until the student is ready to practice alone. Through the support service delivery process, trust is built and sustained within communities as an essential component of public health academic community engagement.

## 1 Introduction

The World Health Organization defines public health “as the science and art of preventing disease, prolonging life, and promoting health through the organized efforts of society” (1). Such organized efforts of society often begin through community engagement defined by the Centers for Disease Control and Prevention (CDC) as “the process of working collaboratively with groups of people who are affiliated by geographic proximity, special interests or similar situations with respect to issues affecting their well-being” (2). Community engagement processes, specifically when initiated or led by public health academics take many forms.

One form public health academic community engagement may take is “outreach,” through which the academic contacts a community in which a public health problem exists (e.g., high smoking and lung cancer rates) to initiate support for a research project on that public health problem (3). Alternatively, academic community engagement may take the form of “consultation” where the academic content expert is called on by a community to share what is known about a particular public health problem (4) (e.g., environmental contamination from specific chemicals and the association with cancer rates). Additionally, academic and public health practice community engagement may also take the form of “collaboration” where public health researchers and members of a community’s local public health system work together to construct a project where goals are shared, and the outcome is implementation of an evidence-based intervention or policy to address community needs (5). Each of these forms of community engagement, involving the public health academic researcher, have the potential to provide elements of community health improvement to the citizens in communities – united by geography, special interests (e.g., aging populations), or similar situations (e.g., lived experience with substance use) (2).

## 2 Forms of academic community engagement

### 2.1 Community based participatory research (CBPR)

The role of the academic, in public health and other disciplines, is typically described through a triad of responsibilities in research, teaching, and service (6, 7). Many public health academic researchers interested in the power of community engagement to create social change conduct Community-Based Participatory Research (often referred to as PAR) (8, 9). Community-Based Participatory Research (CBPR) is defined as “an approach in which researchers and community stakeholders form equitable partnerships to tackle issues related to community health improvement and knowledge production” (5). Steps to CBPR include:

- Establish partnership,
- Define a question of importance to the community,
- Design and execute the research,
- Translate the results into action (5),

CBPR is frequently an amalgamation of community engagement outreach, consultation, and collaboration.

Case example: Using a team of academic and community based researchers, the Appal-TREE project used a CBPR method, structured public involvement, to tailor several healthy eating interventions, conduct, and evaluate those interventions in a specific Appalachian community (10).

Of particular interest for community engagement is the CBPR step of establishing partnerships. Such partnerships are considered foundational for CBPR beginning with the identification of a community collaborator (11) and building trusting relationships (12–14).

### 2.2 Implementation research

Another area of scientific study often used by academic researchers interested in community engagement is Implementation Research. Implementation Research is the scientific study of the use of strategies to adopt and integrate evidence-based health interventions into clinical and community settings to improve patient outcomes and benefit population health (15, 16). Frameworks are used in Implementation Research to understand the interrelated elements of a project that are potential influencers of outcomes (17).

Case example: One implementation study evaluated two strategies, coaching and ECHO sessions, to identify which was more effective to increase buprenorphine use in jail settings (18).

One widely used evaluation framework in implementation research is RE-AIM (19) which includes the following constructs:

R – Who actually participates in the intervention and how will they be reached?
E – Effectiveness-What is/was the most important benefits you are trying to achieve,
A – Adoption-Where is/was the program or policy applied and who applied it?
I – Implementation-How consistently is/was the program or policy delivered,
M – Maintenance – When will/was the initiative become operational; how long will/was it be sustained (19).

The first construct of RE-AIM, Reach, guides the researcher to consider who is to benefit from the intervention being studied and includes the decisions of how the target population will be reached (20). Researchers using RE-AIM will focus on selecting from the a variety of strategies, including “developing stakeholder relationships” to improve the intervention implementation (17, 21–23). Similar to CBPR’s foundational element of building partnerships, implementation researchers must also interact to develop stakeholder relationships whether they are stakeholders who are implementing the evidence-based intervention or those individuals receiving the intervention (15, 16).

Case example: This study utilized the RE-AIM framework to describe implementation of the virtual Happy Healthy Homes intervention, a nutrition and environmental health intervention (24).

### 2.3 Building trusting relationships

Building trusting community relationships is often described as a slow process (12) that may change over time from initiation to a sustained relationship (25). Elements that impact trust include the personal qualities of researchers such as the ability to deliver clear, honest communication (26), strong listening skills, empathy, and respect (27–29). Trust may also be impacted by actions taken in the field such as being visible in the community (26), demonstrating relevant expertise, delivering on commitments, and providing support to community initiatives (27).

Challenges for public health academic researchers to building partnerships based on trusting relationships include the time required to understand a community, the people, and the conditions in which residents live and work (9). This time spent in communities and with community members is time that often includes nights, weekends, and participation in community events and activities (9). Researchers must also realize the past experiences of community members with previous researchers may not have been positive (12). In such cases, community members, particularly in vulnerable communities, many feel “examined” or “exploited” when researchers employed a “helicopter approach” in which they “fly in” to take information but the community sees nothing in return (9, 26). Some literature even cautions researchers about developing relationships that become friendships in communities—suggesting that allowing relationships to develop into friendship might result in hurt feelings when researchers leave the community at the conclusion of specific projects (28). However, this issue may be effectively minimized through the consistent and continual engagement among partners.

Although additional models exist, such as Community Engagement Studios, the relationship with community members is initiated with the goal of improving the research product. This paper will provide a means through which to engage with communities external or prior to a research project.

## 3 Proposal: enhanced academic role in community engagement

The faculty triad, the aforementioned role of the academic, includes research, teaching, and service (6, 7). For those interested in community engagement, this paper has provided introductory information on two research methodologies with community engagement components – CBPR and Implementation Research. While the goals of both methods include working with the community and the development of trusting relationships (5, 30), both methods also indicate that the goals of these relationships are to “tackle issues related to community health improvement” (5) and “improve patient outcomes and benefit population health” (15, 16). Is it possible that there are gaps in the processes described in CBPR and Implementation Research to making a sustainable difference in the health of communities? Literature on CBPR discusses beginning the process with the community through the identification of a collaborator (9). How is this done? How is the relationship with the collaborator initiated? Is there room for improvement in the process of matching communities and researchers? Who has this information? In addition, Implementation Research frameworks state that the researcher must identify how the target population will be reached. Are there new ways that could maximize what we learn through community research methods and the implementation of that knowledge for community health improvement? The purpose of this proposal is to present a method to both assist with trusting relationship development and support communities.

### 3.1 Enhancing the academic role through support service delivery

As in the discussion of CBPR, Implementation Research, and the roles of public health researchers in communities, this proposal will focus on building trusting relationships through support service delivery to the community, not by identifying a new method of community engagement, but through the enhancement or expansion of the academic’s responsibilities. The answer to the question of how we might build trusting community relationships could lie in the last element of the faculty triad – service. Service is often the aspect of the academic responsibilities with the most obscure definition (7). For some academic organizations, service may be those duties that simply do not fall within the research or teaching areas (6). In other organizations, service will greatly depend on the academic home or department a faculty member belongs to indicating different definitions of service for different departments or colleges within the university setting (6). Service might also be defined based on where or with whom the service is done such as service to the university, service to the academic department, service to the profession, and/or service to the community (6).

For the purposes of this proposal, support service delivery will be defined as the provision of services to support community engaged health improvement projects in which the academic’s expertise is delivered as support in the community. The expertise, discussed here, would not be given primarily as a didactic training to public health practitioners, rather through a similar mechanism to preceptor guided training in certain health disciplines in which an expert demonstrates, teaches, and supports until the student is ready to practice alone (31).

Support service delivery could begin the process of becoming known to the community with participation in a community group whose mission matches the area of expertise of the academic. Through this group, the academic becomes known to the community in a non-threatening, mutually beneficial learning scenario. Examples could include community health improvement coalitions, diabetes educator groups, substance use prevention groups, community-based emergency preparedness coalitions and many others. Reaching out to local leaders of these groups for permission to attend meetings and learn from those in practice, without requesting anything in return, could open doors for relationship building and true learning both by the academic and the community members. Academic service provided through support service delivery in communities could include some of the following examples:

- The public health academic whose expertise is in advocacy could support local health department (LHD) staff interested in obtaining support for a health-related policy by showing them how to develop and deliver an advocacy talk at a local civic club meeting by actually delivering the talk with LHD staff in attendance.
- The public health academic whose expertise is in working with health-related data could gather publicly available secondary data at the jurisdiction level and provide not only the data for immediate use and decision making but the location of the data so that the LHD staff could update as needed on their own.
- The public health academic whose expertise is in emergency preparedness could work with communities to practice emergency management plans and/or respond to an actual emergency that has impacted a community.

This idea of delivering the academic’s expertise in communities, separate from seeking community organizational support of a research initiative or recruiting participants for a research study, could be applicable in many disciplines. However, building trusting relationships and working in communities is foundational to public health. The 10 Essential Public Health Services includes the core responsibility of “mobilizing community partnerships” through which the public health professionals collaborate with community groups on a variety of health-related issues (32). Support services delivery provides a means through which community partnerships are mobilized to learn and move forward to address community health improvement.

For example, a public health academic whose expertise is in coalition building could also work with community-facing public health staff to facilitate a coalition group decision-making process as part of a community health improvement planning initiative by providing facilitation services for staff to observe and practice. Another example is the preparation for and actual implementation of evidence-based practice. Many practitioners may not have access to university quality library resources to locate evidence-based interventions for community-identified problems but may also be without experience or knowledge of the key elements of successful implementation. The communities in which these practitioners serve may not have had the benefit of participating in community-engaged academic research techniques, such as CBPR or Implementation Research, but could be open to an academic support service provider demonstrating and educating them on key implementation steps.

Support service delivery provides opportunities for the public health academic to deliver those elements, previously mentioned, as known contributors to building trusting relationships – visibility in the community (26), demonstrating relevant expertise, delivering on commitments, and providing support to community initiatives (27). The time spent in service to communities could help reduce the challenge, previously mentioned in the context of researchers, of time needed “on the ground” to learn about communities and the people who live and work there (9). Service delivered via this proposed approach would be done without expectation of research participation and/or support thus providing a means of changing the image of the academic “helicopter” researcher to be that of a trusted academic partner (9).

## 4 Case study: a support service delivery example in Kentucky: academic and local health department relationships

### 4.1 Background: local health departments

Consider first the 3,000 + local public health departments (LHD) located in communities of varying sizes across the nation. Based on the 2022 Profile of Local Health Departments by the National Association of County and City Health Departments, 62%, of the over 2,500 health departments responding to the survey, serve jurisdictions of fewer than 50,000 people (33). Of the total respondents to the profile survey, an overwhelming majority (81–99%) indicated that they have active partnerships with healthcare and community partners, making the LHD an ideal starting place for community engaged models of health improvement (33). However, since 2013, 60% of LHD top executives have been in their positions for less than five years and for the small health departments (covering a jurisdiction of <50,000) only 28% of those leaders had a degree in public health. As jurisdictions grow in size (large = jurisdiction of 500,000+), the number of leaders educated in public health increases to only 50% (33). These LHD leaders guide a public health workforce that varies greatly in size based on the rural or urban location of the LHD and has nurses (85%) as the most frequently identified discipline in the small health departments, followed by office and administrative staff, environmental and emergency preparedness staff (33).

With the increasing public health concerns of many areas, specifically those in rural areas like the majority of communities in Kentucky, the strain on the LHD staff and members of their community partner systems is great (34–36). From this perspective, the service delivery proposal posits that the missing link in the previously discussed community engagement methods of outreach, consultation, and collaboration is support service delivery.

### 4.2 Academic relationships with local health departments

In 2011, faculty and staff of the University of Kentucky’s College of Public Health (UKCPH), funded by a HRSA Public Health Training Grant, began attending Kentucky Health Department Association meetings, interacting with practitioners and learning from each other. The Public Health Accreditation Board had recently developed the standards and measures of national voluntary public health accreditation, and many Kentucky health departments had no experience with interpreting those standards or engaging their communities in the required Community Health Assessment and Community Health Improvement Plan (CHA/CHIP).

Kentucky health department leaders approached the faculty and staff of UKCPH to discuss means of collaboration to increase local health departments ability to meet accreditation standards. A result of these discussions was the formation of an accreditation learning collaborative and the initiation of support service delivery for accreditation readiness. Faculty and staff of UKCPH received training in the Mobilizing Action through Planning and Partnerships (MAPP) model of the CHA/CHIP process and worked with LHDs to adapt the model for the needs of communities. Some communities had completed the community health assessment but did not know how to proceed with action items to address the identified needs. Utilizing the concept of support service delivery, UKCPH faculty and staff gathered information on evidence-based practices for the most frequently identified needs in Kentucky communities, distributed the information, gathered publicly available data, and facilitated decision making sessions in Kentucky communities. Led by facilitation-trained UKCPH faculty and staff, these decision-making sessions involved working with community groups to gather and synthesize qualitative and quantitative community data. As the trusted academic partner, UKCPH facilitators utilized theoretical knowledge to walk community groups through the decision-making process, ultimately resulting in comprehensive assessment and identification of community health issues.

For the faculty and staff of UKCPH providing support service delivery in Kentucky communities, a number of positive results have been noted. In addition to the Kentucky LHDs actively pursuing and receiving public health accreditation, the relationships formed between local health department leaders, their staff, and the faculty and staff of UKCPH have been active for over ten years, following a low-cost fee-for-service funding model after grant funding concluded. UKCPH faculty and staff have committed to ongoing relationships without any expectation of leaving when a particular project is complete. These trusting relationships have resulted in the introduction of community members to researchers for projects including cancer screening and prevention, diabetes management, substance use prevention and others. In addition, the work of UKCPH faculty and staff includes the formation of an Academic Health Department between one seven county district health department and UKCPH. Through this Academic Health Department structure, UKCPH has delivered staff training, team building exercises, accreditation readiness in the form of CHA/CHIP, strategic planning, and workforce development planning. The health department leadership have also participated with students in classes to explain local health department leadership and management issues and health improvement initiatives such as harm reduction and breastfeeding. Through these continued collaborations, it is important to consider the ever-evolving nature of communities and their health needs. As such, UKCPH academic partners consistently re-evaluate the public health needs and remain equipped to research and address them.

## 5 Discussion

The concept of community engagement, working collaboratively with communities, is recognized as a valuable component of research methodologies particularly those working on health improvement initiatives. Both CBPR and Implementation Research have key elements that involve developing trust and relationships within communities. The “how” of developing trusting relationships is less clear and often difficult due to academic researcher time constraints and previous negative experiences with research in communities. However, this paper proposes utilizing the academic responsibility to provide service through the concept of support service delivery.

Support service delivery provides a means of developing trusting relationships by providing a service through expertise and support without asking for anything in return. For public health academics who deliver these support services, the opportunity to build trusting relationships is enhanced by spending time in the community, learning from community members expertise, demonstrating relevant expertise in issues the community has, and delivering on commitments of support.

For the public health academic who embraces support service delivery to be successful as a team science member providing introductions for other researchers in communities as well as an understanding of the conditions in which community members live and work, additional research is needed on the following elements:

- What are the characteristics of a successful academic who provides support service delivery? Does that academic need to have a history with the community?
- What impact could the concept of support service delivery have on academic service?
- What methods of support must be in place at the college or university for public health academics providing support service delivery?
- Do community members, where support services have been delivered, have a higher trust in the academic, than communities who have not received support service delivery?
- Considering that many communities may never have the opportunity to participate in a CBPR or Implementation Research project, could the academic delivering support services provide dissemination of evidence-based practice? Could this dissemination and support shorten the time from development of evidence-based practice to the acceptance and use of that evidence?

## Data Availability

The original contributions presented in the study are included in the article/supplementary material, further inquiries can be directed to the corresponding author.
